# Comprehensive genome analysis of *Burkholderia gladioli* BGLCN isolated from rice seedlings in Thailand reveals potential for secondary metabolite production

**DOI:** 10.1016/j.dib.2025.112235

**Published:** 2025-11-03

**Authors:** Sujin Patarapuwadol, Pornpavee Nualnisachol, Jiraphan Premsuriya

**Affiliations:** a Department of Plant Pathology, Faculty of Agriculture at Kamphaeng Saen, Kasetsart University Kamphaeng Saen Campus, Nakhon Pathom, , 73140, Thailand; b Princess Srisavangavadhana Faculty of Medicine, Chulabhorn Royal Academy, Bangkok, , 10210, Thailand; c Research Center on Clinical and System Microbiology (RCSym), Chulabhorn Royal Academy, Bangkok, , 10210, Thailand

**Keywords:** *Burkholderia gladioli*, Complete genome, Secondary metabolite, Rice pathogen

## Abstract

*Burkholderia gladioli* is a Gram-negative bacterium with multi-host adaptability as both a plant pathogen and an opportunistic human pathogen. It is also known as a producer of diverse bioactive metabolites. This dataset presents the first complete genome of *B. gladioli* isolated from Thailand, designated strain BGLCN, recovered from rice seedlings in Chai Nat Province. In preliminary assays, this strain exhibited *in vitro* antibacterial activity against human pathogens, including *Acinetobacter baumannii, Escherichia coli*, and *Staphylococcus aureus*, making it a promising candidate for the study of antibacterial compounds. The genome was sequenced using both Illumina NovaSeq and Oxford Nanopore PromethION platforms. A hybrid assembly generated two complete chromosomes and four plasmids with total genome size of 7.93 Mb with a GC content of 67.83 % and 100 % completeness. The genome contains 7266 coding sequences, 15 rRNA genes, and 84 tRNA genes. Phylogenomic analysis showed that BGLCN is closely related to rice-derived isolates from Vietnam. Biosynthetic gene cluster analysis using antiSMASH and DeepBGC revealed several putative secondary metabolite pathways, including polyketides, nonribosomal peptides, and saccharides. This dataset provides a valuable reference for comparative genomics, phylogenetic studies, and exploration of bioactive compound biosynthesis in *B. gladioli*. The genome is available under BioProject PRJNA1337644.

Specifications Table

**Table:** 

Subject	Biology
Specific subject area	Microbiology, Genomics, Bioinformatics
Type of data	Table, Figure, Complete genome sequence in FASTA format Raw, Filtered, Analyzed
Data collection	*Burkholderia gladioli* BGLCN was isolated from a rice seedling in Chai Nat, Thailand, using nutrient agar. Genomic DNA was extracted with the ZymoBIOMICS DNA Miniprep Kit and sequenced using the Illumina NovaSeq and Oxford Nanopore PromethION P24 platforms. Read quality was assessed with FastQC and NanoPlot, while adapters and low-quality reads were removed using Fastp, Porechop, and Filtlong. Hybrid genome assembly was performed with Unicycler, and assembly completeness and contamination were evaluated using CheckM and QUAST. Chromosomal identity and taxonomic placement were confirmed using GTDB-Tk. Genome annotation and biosynthetic gene cluster prediction were conducted with Prokka, antiSMASH, and DeepBGC.
Data source location	Institution: Department of Plant Pathology, Faculty of Agriculture at Kamphaeng Saen, Kasetsart University Kamphaeng Saen Campus City/Town/Region: Nakhon Pathom Country: Thailand GPS coordinates: 14°01′25.34″N 99°58′23.94″E
Data accessibility	The assembly data have been deposited in a public repository, and the analyzed results are described in this report. Repository name: Complete genome sequence of Burkholderia gladioli strain BGLCN, isolated from rice seedlings in Thailand deposited in NCBI. BioProject: PRJNA1337644 BioSample: SAMN52368994 Direct URL to data: https://www.ncbi.nlm.nih.gov/bioproject/PRJNA1337644 https://www.ncbi.nlm.nih.gov/biosample/SAMN52368994/
Related research article	W. Pet–amphai, J. Watcharachaiyakup, S. Patarapuwadol, W. Kositratana, Identification of Bacterial Pathogens Causing Panicle Blight and Dirty Panicle of Rice by Multilocus Sequence Analysis (in Thai), Agricultural Science Journal 48;2 (2017) 297–311.

## 1 Value of the Data

• This dataset represents the first complete genome of *B. gladioli* from Thailand available in the NCBI database.
• The hybrid assembly using both short- and long-read sequencing data generated a high-quality and complete chromosomal and plasmid sequences.
• The complete genome sequence of *B. gladioli* BGLCN could serve as a valuable resource for comparative genomic studies with other *B. gladioli* strains, particularly in the contexts of plant pathology and secondary metabolite research.

## 2 Background

*Burkholderia gladioli* is a versatile Gram-negative bacterium known for its multi-host adaptability as both a plant pathogen and an opportunistic human pathogen. It has been isolated from various ecological niches, including soil, plants, and clinical environments [1,2]. Although *B. gladioli* is generally recognized as a pathogen, some strains can produce a diverse array of bioactive secondary metabolites, several of which have potential pharmaceutical or agricultural applications [[3], [4], [5]]. Despite its importance, genomic data for *B. gladioli* strains from Thailand remain limited, particularly complete genome assemblies that enable detailed comparative analyses. This dataset was generated to provide a high-quality genome for *B. gladioli* BGLCN, isolated from rice seedlings in Chai Nat Province, Thailand [6]. The initial testing suggests this strain is a candidate for investigating antibacterial compounds, as it has shown the ability to inhibit human pathogens *in vitro*, including *A. baumannii, E. coli*, and *S. aureus*. The genome was sequenced using a hybrid approach that combined Illumina and ONT data, ensuring accuracy and completeness. The assembly and annotation provide a valuable resource for exploring genomic diversity and biosynthetic potential among *B. gladioli* strains. This dataset complements ongoing studies on *Burkholderia* genomics and contributes to a broader understanding of the ecological and metabolic versatility of this species.

## 3 Data Description

**Fig. 1:**
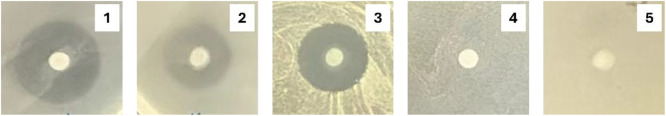
Antibacterial activity of *B. gladioli* BGLCN demonstrated by a soft agar overlay assay. 1 = *Acinetobacter baumannii* ATCC 17,978, 2 = *Escherichia coli* ATCC 25,922, 3 = *Staphylococcus aureus* ATCC 29,213, 4 = *Pseudomonas aeruginosa* ATCC 27,853, 5 = *Klebsiella pneumoniae* ATCC 700,603. Clear zone indicates growth inhibition activity.

**Fig. 2:**
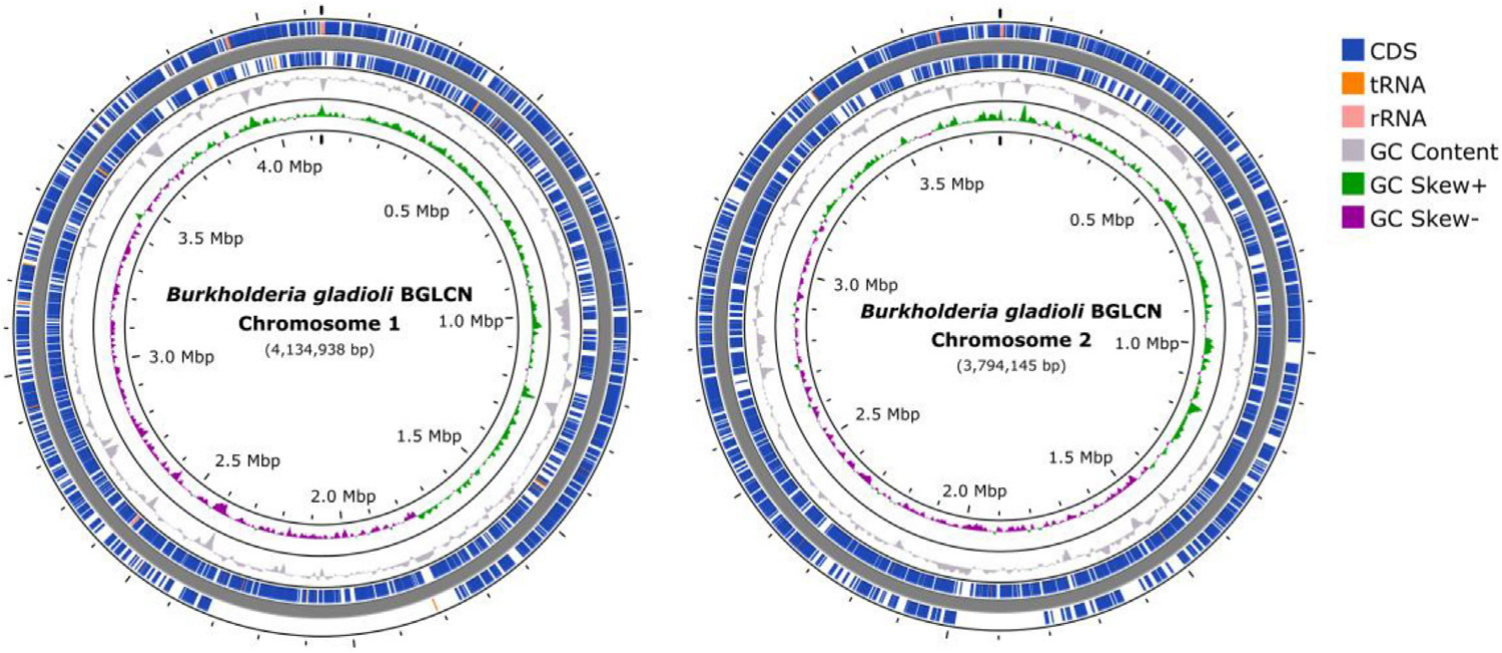
Circular genome map of *B. gladioli* BGLCN chromosome 1 and chromosome 2. From the outermost layer to the center, the map includes CDS and RNA genes on the forward strand and reverse strand, GC content, GC Skew + and GC Skew -.

*B. gladioli* BGLCN was isolated from rice seedlings in Chai Nat Province, Thailand in 2013. Soft agar overlay assay showed that this strain had *in vitro* antibacterial activity against three medically important pathogens, including *A. baumannii, E. coli*, and *S. aureus* (Fig. 1). Whole genome sequencing utilizing both Illumina and ONT yield a high-quality, complete genome. Genomic relatedness analysis based on average nucleotide identity (ANI) with other *B. gladioli* genomes showed a value of 97.92 %, confirming the identification of BGLCN as *B. gladioli*. A summary of the genomic characteristics of *B. gladioli* BGLCN is presented in Table 1 and Fig. 2. Beside chromosomes, four putative plasmids were identified with the size ranging from 114,984 to 174,243 bp.

**Table 1:** Genomic description of *B. gladioli* BGLCN.

Characteristics	Value	Source
Genome Length (bp) ▪ Chromosome 1 (bp) ▪ Chromosome 2 (by)	7929,092 4134,938 3794,154	QUAST QUAST QUAST
Number of contigs (Total) ▪ 2 (Chromosome) ▪ 4 (Plasmid)	6 2 4	QUAST QUAST QUAST
GC content ( %)	67.83	QUAST
Coverage (X)	569	CheckM
Completeness ( %)	100	CheckM
Number of CDS	7266	Prokka
Number of rRNA genes	15	Prokka
Number of tRNA genes	84	Prokka
AIN ( %)	97.92	GTDB-Tk

**Fig. 3:**
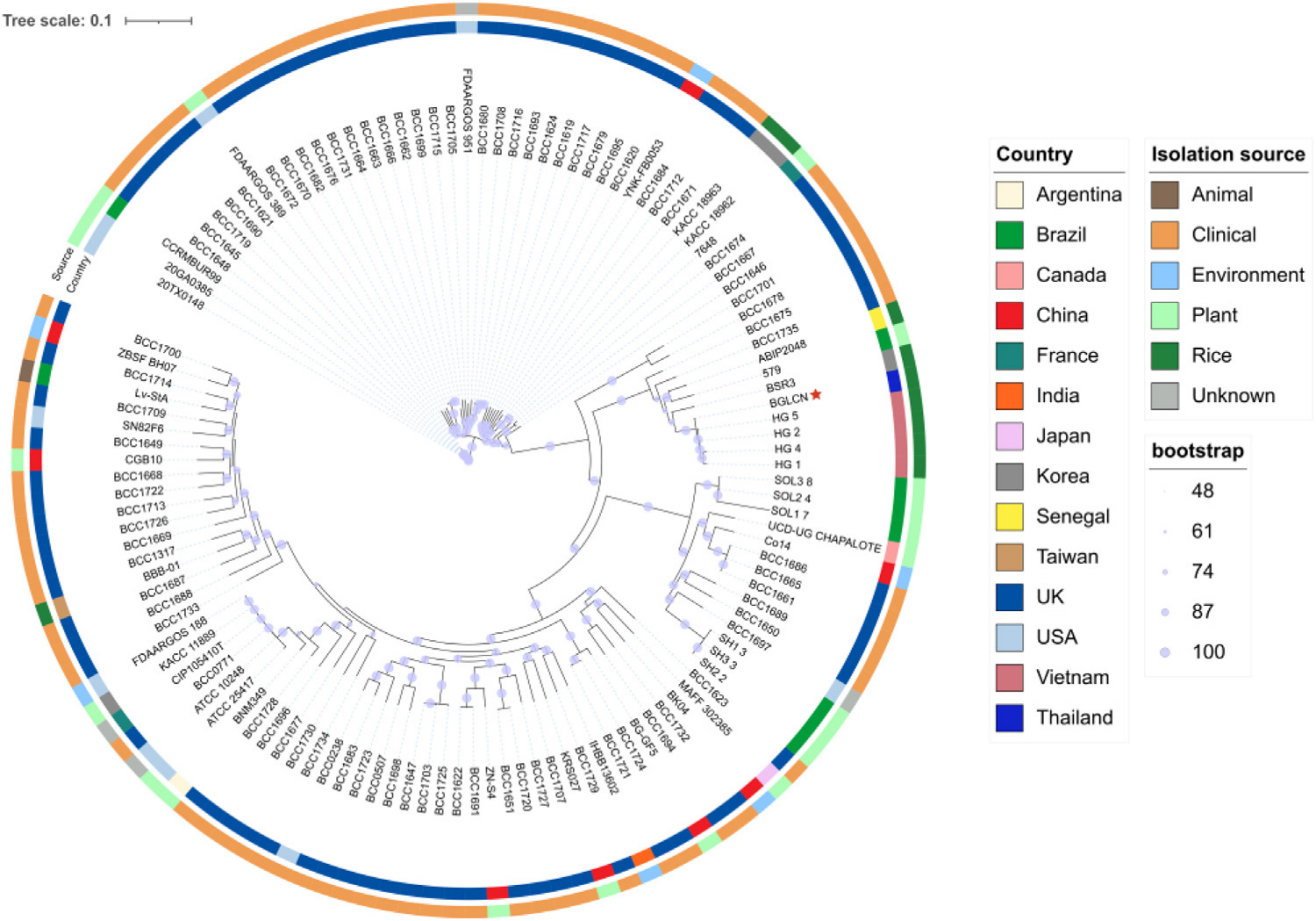
SNP-based phylogenetic tree reconstructed from whole-genome sequence data of *B. gladioli* BGLCN and other *B. gladioli* genomes available at the scaffold assembly level in the NCBI database. Bootstrap support was calculated from 1000 replicates. The tree is annotated with the country of origin and isolation source for each strain.

The evolutionary relationships between BGLCN and other *B. gladioli* genomes available in the NCBI database are illustrated in the SNP-based phylogenomic tree shown in Fig. 3. BGLCN is closely related to rice isolates from Vietnam.

To identify potential bioactive compounds, the genome was analyzed for biosynthetic gene clusters (BGCs) using antiSMASH v7.1.0 [7] and DeepBGC [8]. antiSMASH identified several secondary metabolite BGCs predicted to produce antibacterial compounds such as enacyloxin IIa [9] and minimycin [10], both showing high similarity scores to characterized reference clusters (Table 2). DeepBGC identified several biosynthetic gene clusters (BGCs) predicted to encode antibacterial compounds on both chromosomes and plasmids (Table 3). Most of these clusters were classified as polyketides, saccharides, and nonribosomal peptides (NRPs). Several BGCs exhibited high antibacterial prediction scores and low cytotoxicity, suggesting their potential as promising candidates for antibiotic development.

**Table 2:** Biosynthetic gene clusters (BGCs) predicted by antiSMASH v7.1.0 in *B. gladioli* BGLCN genome.

Location*	Type of BGCs	Most similar known cluster	Additional information	Similarity
**Chro. 2**	T1PKS, transAT-PKS-like	Enacyloxin iia	NRP+Polyketide	100 %
**Chro. 2**	NRPS	Kolossin	NRP	100 %
**Chro. 2**	NRP-metallophore,NRPS	Plantaribactin	NRP	100 %
**Chro. 2**	NRPS	Gladiochelin A1/gladiochelin B2	NRP	86 %
**Chro. 2**	NRPS-like	Minimycin	NRP+Saccharide	80 %
**Chro. 1**	transAT-PKS-like,T3PKS,PKS-like	Isobongkrekic acid/bongkrekic acid	Polyketide:Modular type I polyketide	78 %

⁎ Chro. indicates chromosome.

**Table 3:** Biosynthetic gene clusters (BGCs) predicted by DeepBGC in *B. gladioli* BGLCN genome.

Location*	Cluster length	Proteins	Domains	DeepBGC score	Product activity	Antibacterial score	Cytotoxic score	Product class
**Chro. 1**	31,004	17	61	0.97912	Antibacterial	0.54	0.36	Polyketide
**Chro. 1**	32,406	26	96	0.94849	Antibacterial	0.5	0.41	Saccharide
**Chro. 1**	55,005	45	91	0.92792	Antibacterial	0.56	0.16	Saccharide
**Chro. 1**	49,732	28	62	0.90123	Antibacterial	0.57	0.24	NRP
**Chro. 1**	4586	5	15	0.89281	Antibacterial	0.68	0.23	Polyketide
**Chro. 1**	10,271	7	16	0.78964	Antibacterial	0.66	0.03	Polyketide
**Chro. 1**	1350	1	5	0.75799	Antibacterial	0.75	0.06	Saccharide
**Chro. 1**	8998	8	31	0.75246	Antibacterial	0.63	0.22	Other
**Chro. 1**	951	1	6	0.72763	Antibacterial	0.57	0.22	Polyketide
**Chro. 1**	1074	1	1	0.71943	Antibacterial	0.87	0.06	Other
**Chro. 1**	1545	1	1	0.71517	Antibacterial	0.86	0.06	Other
**Chro. 2**	54,650	20	47	0.95277	Antibacterial	0.5	0.1	NRP
**Chro. 2**	10,131	6	14	0.91855	Antibacterial	0.51	0.04	NRP
**Chro. 2**	18,859	16	39	0.88597	Antibacterial	0.51	0.1	Other
**Chro. 2**	22,040	18	41	0.86635	Antibacterial	0.53	0.13	Other
**Chro. 2**	8316	7	18	0.84378	Antibacterial	0.65	0.05	Saccharide
**Chro. 2**	23,261	5	12	0.83606	Antibacterial	0.68	0.07	NRP
**Chro. 2**	5366	3	9	0.79719	Antibacterial	0.62	0.27	Other
**Chro. 2**	7503	7	18	0.7794	Antibacterial	0.67	0.03	RiPP
**Chro. 2**	3475	4	8	0.77809	Antibacterial	0.59	0.04	Other
**Chro. 2**	9999	7	11	0.7776	Antibacterial	0.57	0.1	Terpene
**Chro. 2**	8100	8	22	0.77711	Antibacterial	0.67	0.14	Polyketide
**Chro. 2**	5678	4	5	0.77385	Antibacterial	0.68	0.18	Other
**Chro. 2**	975	1	5	0.73344	Antibacterial	0.82	0.06	Polyketide
**Chro. 2**	5961	6	10	0.72473	Antibacterial	0.72	0.04	RiPP
**Chro. 2**	1806	1	2	0.70948	Antibacterial	0.87	0.07	Other
**Plas. 1**	6601	4	16	0.77486	Antibacterial	0.81	0.05	Other
**Plas. 1**	2091	2	12	0.76361	Antibacterial	0.73	0.1	Other
**Plas. 1**	4808	5	6	0.73372	Antibacterial	0.88	0.1	Other
**Plas. 1**	10,766	3	10	0.72971	Antibacterial	0.89	0.09	Other
**Plas. 1**	978	1	3	0.70371	Antibacterial	0.8	0.07	Other
**Plas. 1**	903	1	1	0.70151	Antibacterial	0.87	0.06	Other

⁎ Chro. indicates chromosome; Plas. indicates plasmid.

## 4 Experimental Design, Materials and Methods

### 4.1 Bacterial isolation

*B. gladioli* BGLCN was isolated from rice (*Oryza sativa* subsp. indica) seeding with seedling rot symptom using nutrient agar with incubation at 30 °C [6]. The identification was performed at Kasetsart University, Kamphaeng Saen Campus, Thailand. Bacterial stock cultures were stored in 20 % glycerol at −80 °C until use.

### 4.2 DNA extraction and WGS

The *B. gladioli* BGLCN strain was streaked onto nutrient agar plates and incubated at 30 °C overnight. Genomic DNA was isolated using the Genomic DNA Purification Kit (Zymo Research, USA) in accordance with the manufacturer’s instructions, with minor adjustments. Instead of harvesting from liquid cultures, bacterial colonies were scraped from the entire agar plate, and the bead-beating duration was reduced to 3 min [11]. DNA quality and quantity were evaluated using a NanoDrop spectrophotometer (Thermo Fisher Scientific, USA). The extracted DNA was subsequently divided into aliquots for short- and long-read sequencing. Illumina NovaSeq 6000 sequencing (150 bp paired-end reads) was performed by Novogene (Singapore). For ONT long-read sequencing, 50 ng of DNA was used to prepare libraries with the Rapid Sequencing gDNA Barcoding Kit (SQK-RBK114.96, ONT, UK). Sequencing was carried out on a PromethION P24 platform equipped with an R10.4.1 flow cell under standard conditions. Basecalling and read quality assessment were conducted using Dorado v0.7.3 with the SUP model v5.0.0 [12].

### 4.3 Bioinformatic analysis

The quality of Illumina short reads was evaluated using FastQC v0.11.9 [13]. Adapter sequences were removed and reads with quality scores below Q30 were discarded using Fastp v0.23.2 [14]. For Oxford Nanopore Technologies (ONT) reads, quality assessment and adapter trimming were carried out with Porechop v0.2.4 (https://github.com/rrwick/Porechop). The reads were then filtered using Filtlong v0.2.1 (https://github.com/rrwick/Filtlong) to retain sequences longer than 1000 bp and with quality scores above Q10. The quality of the filtered ONT reads was further examined using NanoPlot v1.38.0 [12]. Hybrid genome assembly was conducted using Unicycler v0.4.8 [15], while assembly completeness and contamination were assessed with CheckM v1.2.1 [16]. General assembly metrics were generated using QUAST v5.0.2 [17]. Contigs were aligned to marker genes from the GTDB-Tk database (R214) to confirm chromosomal identity, and average nucleotide identity (ANI) and alignment fraction values were calculated using GTDB-Tk v2.1.1 [18]. Genome annotation was performed with Prokka v1.14.6 [19]. Biosynthetic gene cluster prediction was performed with antiSMASH v7.1.0 [7] and DeepBGC v0.1.23 [8].

Phylogenetic construction was carried out using the *B. gladioli* BGLCN genome together with 123 publicly available *B. gladioli* genomes retrieved from the GenBank database (accessed on 1 October 2025). Only assemblies with scaffold-level or higher completeness were considered for inclusion. Core-genome single-nucleotide polymorphisms (SNPs), encompassing substitutions, insertions, and deletions, were detected using Snippy v4.6.0 (https://github.com/tseemann/snippy). A maximum-likelihood phylogenetic tree was subsequently generated from the concatenated SNP alignment using IQ-TREE v2.4.0 [20]., with the GTR+*G* model and 1000 bootstrap replicates and *B. gladioli* BBB-01 as the reference genome (GCF_016698705.1). The final tree was visualized using iTOL v6 [21].

## Limitations

The biosynthetic pathways for antibiotics and other bioactive metabolites identified in this study are currently based on computational predictions and remain hypothetical. In the absence of experimental confirmation, these predicted pathways should be interpreted with caution, as they may not accurately represent the true biochemical processes operating in *B. gladioli* BGLCN. Future investigations integrating functional genomics and biochemical characterization will be essential to validate these *in silico* predictions and determine their biological relevance.

## Ethics Statement

This study was approved by the Chulabhorn Royal Academy Research Committee. This research excluded human or animal subjects; therefore, ethical approval was not required. The authors declare that this manuscript presents original work that has not been published or submitted for publication elsewhere.

## Credit Author Statement

**Sujin Patarapuwadol:** Conceptualization, Methodology, Supervision, Writing- Reviewing and Editing; **Pornpavee Nualnisachol:** Software, Data curation, Visualization, Writing- Reviewing and Editing; **Jiraphan Premsuriya:** Conceptualization, Methodology, Data curation, Visualization, Writing, Original draft preparation, Writing- Reviewing and Editing.

## Data Availability

Burkholderia gladioli strain:BGLCNBurkholderia gladioli strain:BGLCN Genome sequencing (Original data).
